# Early Experience With Barbed Sutures for Abdominal Closure in Deep Inferior Epigastric Perforator Flap Breast Reconstruction

**Published:** 2012-05-21

**Authors:** Catherine de Blacam, Salih Colakoglu, Adeyiza O. Momoh, Samuel J. Lin, Adam M. Tobias, Bernard T. Lee

**Affiliations:** ^a^Department of Surgery, Division of Plastic and Reconstructive Surgery, Beth Israel Deaconess Medical Center, Boston, MA; ^b^Department of Surgery, Section of Plastic Surgery, University of Michigan Health System, Ann Arbor

## Abstract

**Objective:** Barbed sutures have recently been introduced for closure of surgical incisions. These self-anchoring sutures incorporate evenly spaced barbs in a circumferential distribution along their length, facilitating knotless wound closure and even distribution of tension along the suture line. In this study, we evaluated postoperative complications associated with the use of unidirectional barbed sutures compared with standard sutures for closure of the abdominal incision in deep inferior epigastric perforator flap breast reconstruction. **Methods:** A consecutive series of 142 patients undergoing deep inferior epigastric perforator flap breast reconstruction were identified at a single institution. The abdominal closure in the first 71 patients was performed using standard suture materials. In the subsequent 71 patients, closure was performed using unidirectional barbed sutures. Patient demographics, complications, procedure time, and costs were compared between standard and barbed suture groups. **Results:** Demographic characteristics and comorbidity profiles were similar between the 2 groups. Overall, there was a significantly higher incidence of complications in the standard suture group (17 vs 7 complications,*P* = .0423). Similar rates of wound infection (*P* = .4412), wound dehiscence (*P* = .4934), and seroma (*P* = .1157) were recorded in both groups. Barbed sutures were $ 15.58 more expensive than standard sutures. No significant difference in total length of operation was observed. **Conclusion:** In this study, the utility of unidirectional barbed sutures in deep inferior epigastric perforator flap breast reconstruction has been demonstrated. Barbed sutures may be useful in a broad range of plastic surgery procedures, not only because of their convenience but also based on favorable clinical outcomes.

While the deep inferior epigastric perforator (DIEP) flap is now a well-established standard in breast reconstruction, refinements in technique continue to be reported.^1^^-^5 Having completed the microvascular anastomosis, a further time-intensive aspect of the case is closure of the abdominal wound. Absorbable barbed suture has been reported in the literature as a safe and efficient method of wound closure in body-contouring surgery.^6^^-^8 The barbs on the suture allow tension to be maintained as the closure proceeds and eliminate the need for subcutaneous knotting, thereby decreasing the overall time required for wound closure.

The V-Loc 180 absorbable wound closure device (Covidien, Mansfield, Mass; Fig 1) is a knotless, unidirectional, self-anchoring suture that incorporates evenly spaced barbs in a circumferential distribution along its length.^9^ The polyglyconate suture is made up of absorbable monofilament and comprises a welded loop design that facilitates a knotless anchor at the beginning of the suture line. In this study, we evaluated postoperative complications associated with the use of these unidirectional barbed sutures compared with standard sutures when used for soft-tissue approximation including fascial repair in patients undergoing DIEP flap breast reconstruction. We also calculated the cost of both suturing techniques and the procedure length in each group.

## METHODS

A consecutive series of 142 patients having DIEP flap breast reconstruction was identified at Beth Israel Deaconess Medical Center between August 2009 and April 2011. In all cases, incisions were closed in 3 layers (Scarpa's fascia, deep dermal, and subcuticular). The first 71 patients underwent abdominal closure with standard sutures including approximation of Scarpa's fascia with interrupted 2-0 Vicryl (Ethicon Surgical, Somerville, NJ), deep dermal approximation with 3-0 Monocryl (Ethicon Surgical), and subcuticular closure with 4-0 Monocryl. In the subsequent 71 patients, abdominal closure was completed using 2-0 V-Loc 180 unidirectional barbed sutures for Scarpa's fascia, deep dermal approximation was performed with fewer 3-0 Monocryls, and subcuticular closure with 3-0 V-Loc 180. Aside from the closure, both groups in the study were managed using the same intraoperative techniques and postsurgical protocols. Each patient had 2 drains placed in the abdominal wound, and these were removed with less than 30-mL fluid drainage over a 24-hour period. The skill level of the fellows and residents working with the senior authors remained consistent throughout the study period.

For all patients, a retrospective review of operative and clinical notes was undertaken. Demographic variables including age and body mass index were recorded, as well as existing comorbidities and incidence of complications in the first 30 days following surgery. Fisher exact or χ^2^ tests and the Student *t* test were used to calculate the statistical difference between groups of dichotomous and continuous variables, respectively. *P* < .05 was considered to represent a significant difference.

Looking at unilateral DIEP flaps only, mean overall operation time was compared between the standard (*n* = 34) and barbed suture (*n* = 39) groups. Cost analysis was performed to compare institutional charges for the sutures in the 2 approaches.

## RESULTS

Demographic characteristics and comorbidity profiles were similar between the patients whose wounds were closed with standard sutures and those in whom barbed suture was used (Table 1). Short-term complications (<30 days) were compared between the standard and barbed suture groups (Table 2). Overall, there was a significantly higher incidence of complications in the standard suture group (17 vs 7 complications, *P* = .0423). Similar rates of wound infection (*P* = .4412), wound dehiscence (*P* = .4934), and seroma (*P* = .1157) were recorded in the 2 groups. Power calculation confirmed adequate sample size for overall complication rate.

A cost comparison was also carried out (Table 3). The institutional charge for the standard suture closure was $38.43 compared with $54.01 for the barbed suture closure. For unilateral DIEP flap procedures, there was no significant difference in mean procedure time between the standard (7 hours 4 minutes) and barbed (7 hours 17 minutes) suture groups (*P* = .5459).

## DISCUSSION

Barbed sutures are one of a number of technologies that have been adopted by plastic surgeons to improve the efficiency of incision closure. As the use of such devices increases, it is important that their efficacy and safety be evaluated for each procedure and application. In this study, we have demonstrated excellent outcomes with absorbable unidirectional barbed sutures for repair of abdominal fascial defects in DIEP flap breast reconstruction.

A significantly lower incidence of postoperative complications was recorded in the barbed suture group. Overall, there were 17 (23.9%) complications in the standard suture group and only 7 (9.9%) in the barbed suture group. Complications in the barbed suture group comprised wound infections (*n* = 2), wound dehiscence (*n* = 3), seroma (*n* = 1), and hematoma (*n* = 1). Only one previous study has looked at the incidence of complications when using barbed sutures in breast reconstruction.^10^ In contrast to our findings, Jandali et al^10^ observed more episodes of delayed wound healing in their barbed suture group. The suture in question was the bidirectional Quill barbed suture (Angiotech Pharmaceuticals, Inc, Vancouver, British Columbia, Canada), and both breast and abdominal wounds were included in the study, however.

The use of barbed sutures was initially validated in minimally invasive face lifting, where the purchase of the barbs opposes the direction of pull and anchors the tissues in place to maintain suspension.^11^ Complications that have been reported in this context include suture extrusion and palpability as well as infection and suboptimal cosmetic results.^11^^-^13 Several previous studies, which looked at both abdominoplasty and Pfannenstiel incision closure, have recorded no difference between standard and barbed sutures in terms of complications.^7^^,^^8^^,^^14^^,^^15^ In contrast, Shermak et al^6^ examined body-contouring wounds from a number of different body sites and recorded a trend toward higher rates of wound complication in their barbed suture group, particularly with regard to brachioplasty closures. A possible explanation for this may be the differing comorbidity profile of our breast cancer population and a cohort of patients with a history of massive weight loss (eg, metabolic syndrome, diabetes, hypertension). Furthermore, more tissue may be removed in postbariatric abdominoplasty than in an average DIEP flap procedure, resulting in a higher degree of tension across the closure, which may account for a trend toward higher incidence of wound problems. These varying reports highlight the importance of carrying out separate investigations for each patient group, procedure type, and body site for which barbed sutures—or indeed any new wound closure device—are being used.

In terms of cost, we found the barbed suture closure to be $15.58 more expensive than closure with a standard interrupted technique. Although these figures are likely to vary between different institutions, they are useful in assessing the relative difference in cost between the 2 techniques. Having demonstrated a significant difference in the number of complications arising from barbed sutures, we feel that this additional cost is minimal and justified.

Decreased procedure time is a commonly cited advantage of barbed sutures and may also offset the additional cost of their use.^6^^-^8^,^^10^ Warner and Gutowski^7^ noted a significant decrease in plication time of abdominoplasty flaps when using the bidirectional Quill barbed suture. A similar observation was made by Rosen,^8^ whose operation length for the same procedure decreased by 15 minutes when the bidirectional barbed suture was used. In the current study, there was no significant difference in procedure length observed between the 2 groups. The likely explanation for this is that the abdominal wound closure in a DIEP flap at our institution is usually performed by an assistant while the microsurgery is in progress. As a result, the precise length of time for this portion of the procedure in isolation is not recorded in the operative log. It is our impression, however, that abdominal closure is indeed quicker with the unidirectional barbed suture. As no difference in the length of time that the patient spends on the operating table can be attributed to the use of the barbed suture, this observation alone is not an indication for the use of the device in the context of free flap breast reconstruction.

Limitations of this study include the relatively small number of patients included and the retrospective nature of the investigation. The next logical step in validating the utility of barbed sutures in autologous breast reconstruction would be a randomized study directly comparing standard and barbed suture closures. Longer follow-up and an assessment of patient satisfaction would also be beneficial.

## CONCLUSION

Barbed sutures are a safe and efficient adjunct to traditional wound closure techniques. In this study, closure of the abdominal incision in DIEP flap breast reconstruction resulted in a lower incidence of overall complications. The use of barbed sutures may be useful in a broad range of plastic surgery procedures, not only because of their convenience but also as a consequence of the favorable outcomes.

## Figures and Tables

**Figure 1:**
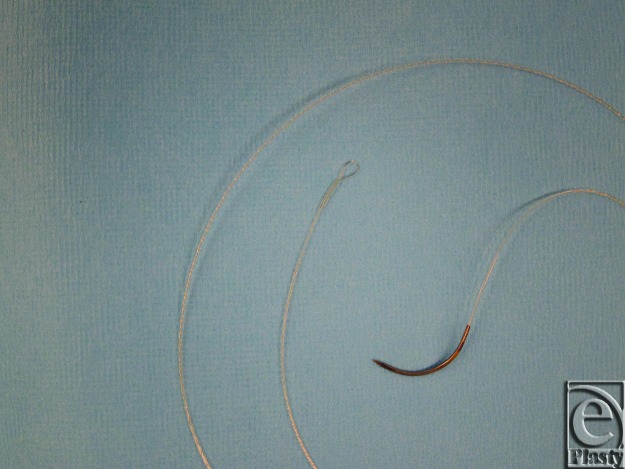
Unidirectional barbed suture.

**Table 1:** Patient characteristics

Demographics	Barbed suture (*n* = 71)	Standard suture (*n* = 71)	*P*
Age, mean	51.2	51.6	.5511
Body mass index average	27.1	27.8	.5785
Laterality			
Unilateral	39	34	.4012
Bilateral	32	37	
Previous abdominal incisions	8	15	.1708
Comorbid conditions			
Diabetes mellitus	3	6	.4934
Hypertension	17	14	.6850
Tobacco use	1	3	.6196

**Table 2:** Complications

Complication	Barbed suture (*n* = 71)	Standard suture (*n* = 71)	*P*
Wound infection	2 (2.8)	5 (7.0)	.4412
Wound dehiscence	3 (4.2)	6 (8.4)	.4934
Seroma	1 (1.4)	6 (8.4)	.1157
Hematoma	1 (1.4)	0 (0)	.0001
Total	7 (9.9)	17 (23.9)	.0423

**Table 3:** Cost comparison

Suture material	Cost, US $
Standard suture	
2-0 Vicryl* × 8	11.19
3-0 Monocryl* × 4	18.16
4-0 Monocryl* × 2	9.08
Total	38.43
Barbed suture	
2-0 V-Loc† × 1	20.44
3-0 Monocryl* × 2	9.08
3-0 V-Loc† × 1	24.49
Total	54.01

*Vicryl and Monocryl (Ethicon Surgical, Somerville, NJ).

†V-Loc (Covidien, Mansfield, Mass).
